# Physical activity patterns in urban neighbourhood parks: insights from a multiple case study

**DOI:** 10.1186/1471-2458-14-962

**Published:** 2014-09-17

**Authors:** Gavin R McCormack, Melanie Rock, Kenda Swanson, Lindsay Burton, Alessandro Massolo

**Affiliations:** Department of Community Health Sciences, Cumming School of Medicine, University of Calgary, 3280 Hospital Drive, N.W, Calgary, AB T2N 4Z6 Canada; Institute for Public Health, University of Calgary, Calgary, AB Canada; Department of Ecosystem and Public Health, Faculty of Veterinary Medicine, University of Calgary, Calgary, AB Canada

## Abstract

**Background:**

Many characteristics of urban parks and neighbourhoods have been linked to patterns of physical activity, yet untangling these relationships to promote increased levels of physical activity presents methodological challenges. Based on qualitative and quantitative data, this article describes patterns of activity within urban parks and the socio-demographic characteristics of park visitors. It also accounts for these patterns in relation to the attributes of parks and their surrounding neighbourhoods.

**Methods:**

A multiple case study was undertaken that incorporated quantitative and qualitative data derived from first-hand observation in a purposive sample of four urban parks. Quantitative data, based on direct observation of visitors’ patterns of use and socio-demographic characteristics, were collected using a structured instrument. Differences in frequencies of observed activities and socio-demographic characteristics of visitors were compared between the four parks. Qualitative data, based on direct observation of park characteristics and patterns of use, were generated through digital photography and analyzed through captioning. Quantitative data on patterns in activity and socio-demographic characteristics were synthesized with the qualitative data on park and usage characteristics.

**Results:**

A comprehensive portrait of each park in the study was generated. Activity types (sedentary, walking, dog-related, cycling, and play), patterns of park use (time of day, day of week), and socio-demographic characteristics (age group, social group) differed between the four parks. Patterns in park use and activity appeared to be associated with socio-demographic characteristics of the surrounding neighbourhoods as well as the physical and social environmental characteristics specific to each park.

**Conclusions:**

Both park and neighbourhood characteristics influence patterns of use and physical activity within parks. The study findings suggest that socio-demographic characteristics of neighbourhoods surrounding parks need be considered in planning, development and management. Engaging local communities could help planners to develop and update urban parks in ways that reflect the needs and characteristics of community residents and, in turn, encourage visits and more physical activity amongst visitors.

**Electronic supplementary material:**

The online version of this article (doi:10.1186/1471-2458-14-962) contains supplementary material, which is available to authorized users.

## Background

Urban settings include a diversity of characteristics that, on the one hand, can be hazardous to human health (e.g., exposure to pollution, motor vehicle traffic, and crime), while on the other hand, can provide health-supportive functions (e.g., sanitation, high-quality drinking water, the experience of nature, and opportunities for social interaction and physical activity) [1, 2]. In particular, urban parks are important for population health because they can impact human health via multiple pathways [3–5] and they can serve as a publically-available recreational facility for many people over multiple generations. Urban parks provide opportunities for socializing, contemplation, and physical activity. Moreover, urban parks can be designed and managed to link people with each other and with nature across a range of different demographic, socioeconomic, and cultural backgrounds [6].

The importance of parks for enhancing the quality of human life has long been recognized. Indeed, urban planning practices since the late 1800s have included provision for park space so as to separate industrial and residential land use, and to serve as central gathering places for civic activities [6]. More recently, local or municipal governments have devoted much of their services and significant proportion of financial resources in the creation, renewal, and upkeep of parks within urban settings. These investments, furthermore, often depend on transfers of resources from higher levels of government and engagement of citizens as tax-payers and volunteers. The provision of functional, attractive, and safe park space is considered to be an important characteristic of healthy communities and cities [7, 8]. Despite their recognized importance, evidence regarding which characteristics of urban parks influence behaviour, in particular physical activity, is only just beginning to be systematically collated and analyzed from the perspective of promoting population health [9–11].

Regular participation in physical activity, especially at recommended levels, can reduce the risk of cardiovascular disease, diabetes, depression, obesity, and some cancers [12, 13]. Moreover, physical activity in urban parks and neighbourhoods can promote social connectedness and interaction, which are also positively linked with physical and mental health outcomes [14, 15]. Existing quantitative and qualitative evidence suggests that parks support physical activity, but not uniformly across the life course [9–11]. Youth activities in parks often involve participating in unstructured activities such as play [16, 17] while for adults of different ages, walking is a common park-based physical activity [18]. Sedentary behaviour (e.g., sitting on a park bench) is also a common park-based activity [19], which may entail walking to, within, and from the park [20]. Variation in the patterns and levels of park-based activity are likely influenced by the interaction between the characteristics of park users and characteristics of the park environment, as well as by the built and social characteristics of the surrounding neighbourhood [21–23].

Bedimo-Rung et al. [24] offer a conceptual framework for understanding how park characteristics influence physical activity, and this framework points to a need for synthesizing data derived from multiple methods. Yet most studies do not explore reasons why such relationships exist or integrate any qualitative data; rather, they focus on the influence of built environmental features on physical activity by adjusting statistically for the influence of other contextual and visitor characteristics [10, 11]. An approach that incorporates both quantitative and qualitative approaches to investigate the determinants of park-based physical activity may provide greater insight into nuanced interactions between park visits, physical features, and socio-demographic characteristics. In addition, whereas qualitative as well as quantitative data about park use can be generated *in situ*[6], qualitative studies of physical activity in urban parks have been dominated by face-to-face and focus group interviews conducted off-site [9]. Thus, for this multiple case study of four urban parks in a Canadian city, we apply quantitative and qualitative methods of direct observation to study patterns of park-based physical activity. The objective of this study was to document and account for patterns of park-based physical activity in ways that could meaningfully inform urban planning and policies to promote health. The University of Calgary Conjoint Human Research Ethics Board granted ethics approval for this study.

## Methods

### Study background and design

Case studies seek to account for the existence of phenomena and the impact of events within specified contexts. Thus, case study designs tend to involve non-probability samples. Each selected case has the potential to yield a great deal of information on its own and in comparison with other cases. This multiple case study emphasized description, using qualitative and quantitative methodological approaches to exploit different data sources in order to generate insights through multiple disciplinary perspectives.

### Sampling strategy

Purposive sampling was used for selecting parks [25]. The sampling strategy was informed by: 1) the recent implementation of a new municipal policy framework for off-leash dog areas, which included designating additional off-leash areas in existing parks [26], and; 2) the larger project, the aim of which was to investigate longitudinal changes in park use, and park-based physical activity and social interaction before and after off-leash designation. We selected the first three existing parks to be publicly-announced as candidate sites for new off-leash areas (West Hillhurst, Meadowlark, and Martindale parks) [26] as well as an approved off-leash park area already in development on the city’s urban fringe (Taradale park) [27]. This paper presents the findings regarding the physical and social environmental characteristics and usage patterns of the four neighbourhood parks two months before final decisions was announced about whether they would become officially designated as sites for off-leash areas.

### Data collection

#### Direct observation: quantitative data

During an initial visit to the four parks (May 2011), observation points were identified and decided upon through consensus by three of the authors (GM, AM, MR). The chosen observation points were centrally located and provided the most comprehensive view of the park area as well as clear sight lines to park entrances and activity settings (e.g., playgrounds, paths, open areas, sitting areas). Only one observation point was necessary for West Hillhurst, Taradale, and Martindale, while two observation points were necessary for Meadowlark because of an elevated section of open space that could not be adequately observed from the other observation point.

From May to July 2011, trained observers stood at the pre-determined observation points within each park and collected data. Data were collected in each park from 830-1230 hrs and 1430-1830 hrs on one weekday (Tuesday or Thursday) and one weekend (Saturday and Sunday) only. Time periods were intended to capture early morning and late afternoon (after school and work) activities; the period from 1230-1430 hrs was not captured because of limited resources. Three observers were rotated between the four parks. Captured user characteristics included gender, age group (i.e., child, teenager, adult, senior), group membership (i.e., at park alone or with others), number of dogs, dog size (i.e., small, medium, large), whether or not dog litter was cleaned up, and activities (Table 1). Each park user was observed for a maximum of two minutes to capture the scope of activities being undertaken and to maximize the number of users that could be observed. Observers selected users by systematically scanning the observation area in an anti-clockwise direction and selecting the next user in the field of vision who had not already been observed. Inter-rater reliability of the structured direct observation was tested at a heavily used park site, not included in the current study, and involved the observation of 72 park users by five observers — three observers of which collected data in the four study parks. The three observers were undergraduate students in the health science or wildlife ecology disciplines; all three received training for this project and had prior experience collecting field data. Variables that had moderate-to-high inter-rater reliability only (i.e., gender, age group, primary and secondary activity, being with other people and being with or without a dog; kappa statistic = 0.52-0.90 and percent of overall agreement = 83.2-95.6%) were collected in the four study parks (Table 1).

**Table 1:** **Inter-rater reliability results for the systematic park observation within a single park (n = 5 raters and 72 park users observed)**

	Kappa*	95% CI	Percent of overall agreement
Gender	0.890	0.786, 0.993	94.7
Age group	0.714	0.595, 0.833	86.4
Primary activity	0.831	0.789, 0.874	86.4
Secondary activity	0.521	0.334, 0.708	83.2
Person belong to a group	0.894	0.805, 0.983	95.0
Any dog with person	0.905	0.814, 0.996	95.6
Cleaned-up dog litter**	0.878	0.786, 0.971	94.2
Size of dog (small, medium, large)**	0.563	0.442, 0.684	71.3

*The estimates are based on average values across pairs of raters [28].

**Based on n = 15 observations identified by all raters as having one dog.

#### Direct observation: qualitative data

Throughout the research process, qualitative data collection was integrated into fieldwork. Two field assistants (KS, LB), who both had prior training in social research on physical activity, compiled a comprehensive photographic record of the parks. The photographs were taken to illustrate attributes of the parks that had the potential to influence patterns of park usage and physical activity. Previous research [9, 24] relevant to the study, and first-hand experience of the field researchers, informed their decisions about which attributes to photograph. The photographs were all taken using a Panasonic DMC-TS3 digital camera, which time-stamps, date-stamps, and assigns global positioning coordinates to each photograph. The photographs were not taken in conjunction with the quantitative direct observations, so as to minimize the potential for distraction and influencing the behaviours of park visitors. KS and LB also provided oral and written reports to MR and GM throughout the fieldwork period, including timely descriptions of critical incidents. For our purposes, critical incidents comprised events and circumstances that interfered with the task of fieldwork and that could, through description and discussion, generate insights relevant to our research objectives. The photographs, and written and oral reports were included in the qualitative dataset maintained using NVivo 10 (QSR International).

#### Additional data sources

To supplement the qualitative and quantitative data derived from direct observation, we inventoried park attributes, consulted published maps, and drew on socio-demographic profiles of the surrounding neighbourhoods and the city as a whole [29]. Neighbourhood socio-demographic characteristic profiles were extracted from the most up-to-date information at the time of analysis (i.e., 2006 Canadian Census and 2011 Calgary Civic Census data). In addition, team members with previous training and professional experience in qualitative research attended public consultations on the potential off-leash designations which took place during the fieldwork period. Participant observation at the public consultations provided the basis for extended fieldnotes and allowed us to collect City-sponsored brochures and reports that were distributed during these events (data not shown). Reports on the results of the consultation sessions posted online, provided information on local people’s perceptions of the parks [26]. These documents were added to the NVivo qualitative dataset.

### Data analysis

This study employed three main analytic strategies; relying on theoretical propositions, developing case descriptions, and examining rival explanations [29]. Analytic strategies in case study research include detailed descriptions that integrate multiple sources of information, pattern-matching across data sources, time-series, and comparisons within a single case and across multiple cases. These analytic strategies tend to be guided by theoretical propositions distilled from the existing literature, and throughout a given project, the research team seeks to substantiate, refine or refute their theoretical propositions [29].

#### Theoretical propositions

For this study, Bedimo-Rung et al.’s [24] conceptual framework was used as a guide. This framework outlines how characteristics of urban parks and surrounding neighbourhoods influence visits and physical activity within parks, to the extent of plausibly influencing health outcomes in populations. Whereas Bedimo-Rung et al. [24] derived this framework by synthesizing quantitative research, this framework is also applicable to qualitative research [9]. According to the framework, important park characteristics include physical features, the condition of these features, accessibility, aesthetics, objective and subjective aspects of safety, and policies. However, our analytic approach treated the other park attributes identified by Bedimo-Rung et al. [24] as manifestations of policy (including a lack of policy or incomplete implementation of policy), rather than treating policy as a discrete category. Our approach also recognized the potential for overlap across these characteristics, and treated instances of overlap as points of entry into complex relationships rather than trying to isolate characteristics as though they were discrete variables.

#### Developing case descriptions

The purpose of developing case descriptions was to specify the activities observed in each of the parks, and to put these observations into context [29]. The techniques of pattern-matching and time-series [29] were employed using the quantitative direct observation data, which involved comparing frequencies of activity, types of visitors, and site characteristics in the four parks. Pearson’s chi-square was used to detect significant differences in observed activities (sedentary, walking, dog-related physical activity, running, cycling, and play activity), gender, age, group association, presence of a dog, time of day, and day of week by park site. Where statistically significant park differences were found based on the Pearson’s chi-square tests (p < .05), Bonferroni adjusted z-tests of proportions was then used to identify significant pairwise differences between parks. Statistical analysis was undertaken using SPSS 19 (IBM Inc.).

As for qualitative data analysis, the techniques of pattern-matching, time-series, and explanation-building were deployed [29]. Following the completion of fieldwork, KS and LB verified geo-codes for each photograph, correcting the global positioning coordinates manually as required on a map of each park. Next, the photographs (including their geo-codes) were imported into NVivo 10, and then sorted by park and by date. Photographs were then captioned in a three-step process. First, the photographs were titled to reflect the focal element of the photograph. Second, keeping in mind the research objectives and based on recollections of the fieldwork from KS, LB or both, the photographs were described in as little as one sentence or as much as paragraph. This phase of captioning also allowed for a time-series approach to analysis, in that photographs could be tagged as being part of a series from a single day or across multiple days (e.g., regular visitors). Third, each description was classified and elaborated upon in relation to park attributes outlined by Bedimo-Rung et al. [24]: features, condition, access, aesthetics, and safety. Here, the emphasis was on explanation-building [29]. Recognizing that citizens view parks in different ways, the captions included different viewpoints that were generated through discussion within the research team.

Qualitative and quantitative data were synthesized, first in the course of developing a descriptive profile for each park and then by comparing across qualitative and quantitative data for all four parks in the study. Analysis took place iteratively; rival explanations were generated and discussed with stakeholder organizations and within the team [29]. In particular, the extent to which observable within-park attributes can account for patterns of activity in a park was continually questioned. Therefore, we actively considered whether the surroundings were just as or even more important than what is or is not found within a specific park (e.g., neighbourhood design, connections roadways and pathways, resident’s socio-demographic characteristics). City documents, fieldwork reports, and debriefing notes were used as supplemental resources to assist with synthesizing the quantitative and qualitative data based on first-hand observation.

## Results

### Descriptions of park and neighbourhood characteristics in relation to patterns of use (qualitative observations)

#### Taradale park

The neighbourhood of Taradale was established in 1984, has a curvilinear street pattern (i.e., cul-de-sacs), and is located in the north east quadrant of the city. In 2011, the neighbourhood consisted of 16,110 residents (Table 2). It has a median annual household income of $71,401 with 15.9% of residents living in low-income households and 9.1% renting. Of those ≥25 years of age, 3.9% are unemployed and 54.8% have less than or equivalent to a high school level education. Over one-third (34.9%) of neighbourhood residents are infants or youth and 60.6% are adults. Fewer than 5% of neighbourhood residents are seniors. Almost half of the Taradale population are immigrants (47.8%), of which two thirds (66.4%) were born in India or Pakistan and one-tenth (9.9%) born in the Philippines (Table 2). Taradale Park was new, and an off-leash area had already been planned and approved at the time of the study. At the public consultation on the potential designation of an off-leash area within the nearby park in Martindale, a couple of participants indicated that they looked forward to using the new off-leash area in the Taradale park [30].

**Table 2:** **Community level sociodemographic characteristics derived from the 2006 Canadian Census and 2011 Calgary Civic Census**

	Taradale % or (count)	Martindale % or (count)	West Hillhurst % or (count)	Meadowlark % or (count)	Calgary % or (count)
**Total population** ^**1**^	(16,110)	(12,987)	(5,757)	(605)	(1,090,936)
**Population mobility** ^**2**^	25.8	19.8	24.0	18.0	20.4
**Rental property** ^**2**^	9.3	12.6	41.0	17.3	27.2
**Age groups** ^**1**^					
Infant/toddler (0–4 yrs)	10.8	9.2	7.0	6.1	6.3
Youth (5–19 yrs)	24.1	22.8	12.5	13.1	17.7
Adult (20–64 yrs)	60.6	63.2	72.2	64.1	66.4
Senior (≥65 yrs)	4.4	4.9	8.3	16.7	9.8
**Low-income households** ^**2**^	14.5	18.1	15.9	6.6	14.2
**Unemployment among those over 25 years of age** ^**2**^	3.9	2.5	1.4	2.9	3.2
**Highest level of education** ^**2**^					
High school or less	54.8	58.3	31.5	36.9	42.7
University (≥bachelor degree)	15.3	13.4	41.1	30.1	25.3
**Population diversity** ^**2**^					
Aboriginal	1.3	3.9	1.7	0.0	2.5
Immigrant	47.8	39.4	13.0	24.8	24.8
Visible Minority	62.6	51.3	8.5	9.9	23.7
**Immigrant population by select countries** ^**2**^					
India	39.5	53.0	0.0	0.0	10.4
Philippines	9.9	7.9	0.0	66.7	10.1
Pakistan	26.9	8.2	0.0	0.0	7.1
United States	0.0	0.0	26.3	0.0	3.0

^1^Data source: 2006 Canadian Census; ^2^Data source: 2011 Calgary Civic Census.

Note: 2006 Canadian Census data are presented as 2011 Canadian Census data are currently not available for all characteristics above or these data are not collected as part of the Calgary Civic Census.

Taradale Park is located on the edge of the neighbourhood and borders a major road (i.e., Stoney Trail) on one side. The park is large (21.59 ha), un-maintained, ‘natural’ field landscape without trees. A baseball diamond was adjacent to the park but separated by a fence. The open green spaces dissected by the dual-use path were undeveloped and overgrown with tall weeds and grass and provided an ideal location for off-leash dogs to run and explore (Additional file 1). The park included a multi-use path that ran through the park and connected to a path running along the park’s perimeter – linking the surrounding neighbourhoods. An informal path ran parallel to the formal path along a wooden fence separating the park from a residential area. This pathway system was frequently used by both pedestrians and cyclists. The formal and informal paths ran past a pond located in one corner of the park. The un-landscaped pond area was populated by a variety of wildlife (e.g., ducks, birds, gophers), and it provided a storm water drainage for the area. This pond was also an attractive feature for larger off-leash dogs, and thus, for the people accompanying them (Additional file 2). Hawks were frequently sighted in the vicinity of this park.

A lack of maintenance (e.g., tall grass overgrown with weeds) made visibility problematic and served as an ideal breeding ground for mosquitos. Household garbage and debris (e.g., drywall and mattresses) ‘dumped’ in the park posed a safety concern for wildlife and visitors such as dogs and children, who might have come into direct contact with the material (Additional file 3). A bench and garbage can surrounded by a small landscaped area was located at one entrance to the park. There were signs at the main entrance to the park and at the seating area to indicate that the park contained an off-leash dog area. Graffiti was evident on the wooden fence at the end of the park at the main entrance adjacent to the residential area. A designated ‘rest stop’, located alongside the main pathways, provided a bench for seating; receptacles for garbage and recycling; and signage (e.g., with directions to areas within and nearby the park, including indication of a designated off-leash area). Older adults of South Asian ancestry, sometimes accompanied by children, would often walk through the park with the ‘rest stop’ as their destination.

#### Martindale park

Martindale was established in 1983, has a curvilinear street pattern (i.e., numerous cul-de-sacs), and is located in the north east quadrant of the city. In 2011, Martindale’s population was 12,987. It has a median annual household income of $65,185 with 18.1% of persons living in low-income households and 12.6% renting their dwellings. Among adults at least 25 years of age, 2.5% are unemployed and over one-half have less than or equivalent to a high school education. A quarter of Martindale residents are youth (22.8%) and 4.9% are seniors. In Martindale, 39.4% of people are first-generation immigrants, of whom 53.0% were born in India, 8.2% were born in Pakistan, and 7.9% were born in the Philippines (Table 2).

Martindale Park is a large open green space (2.48 ha) which includes a natural wooded area in one corner and trees along the park perimeter. A paved pathway ran through the park and exited onto the streets of the surrounding neighbourhood on either side. Dual-use paths ran through the park and an informal path ran through the natural wooded area (Additional file 4). An open grassy area containing a storm water drainage basin provided an open space for dogs to run and play off-leash, however; it was often flooded and maintenance (e.g., mowing) was often incomplete, contributing to a ‘mosquito problem.’ The park had no benches or seating nor did it have any dedicated lighting (Additional file 5). A garbage can was located beside a formal path which was adjacent to street parking allocated for park visitors. An unkempt wooded area existed alongside the paved path at the rear of the park (the nearest that this park came to any of the houses in the neighbourhood). The interior of this wooded area was not visible unless one walked directly inside (i.e., through the bushes); it was overgrown with weeds, covered with litter, and frequently visited by youth (who would often arrive by bicycle and stay within the wooded area for long periods of time). The dog-walkers who regularly visited this park (at least once or twice daily) tended to be white males. South Asian and Southeast Asian visitors tended to be adults who walked briskly through the park. On occasion, adults brought school-aged or preschool-aged children to the park, but they did not tend to stay for long or return on a regular basis. Whereas a public art installation located at the front of the park is suggestive of a playground, the park does not contain a playground. The public art installation consists of three brass figures; a Caucasian-looking woman pushing a girl on a swing-set and an off-leash Cocker Spaniel jumping in the grass nearby. In public consultations regarding the potential designation of an off-leash area within this park, local residents indicated that this park is a much-needed amenity but they also cited concerns about litter and safety (within the park and from traffic) [30].

#### West Hillhurst park

West Hillhurst was established in 1945, has a grid street pattern with streets that are lined with mature landscaping and sidewalks on both sides, and is located in the northwest quadrant of the city. In 2011, West Hillhurst’s population was 5,757 (Table 2). The majority of West Hillhurst residents were born in Canada, however; the socio-economic status is mixed, and a process of gentrification was apparent from the housing, gardens, and yard maintenance. The community has a median annual household income of $61,401, with 15.9% of the neighbourhood residents living in low-income households, 31.5% having less than or equivalent to a high level education, and 1.4% unemployment. The proportion of West Hillhurst residents dwelling in rental properties is high (41%) compared with the Calgary overall (27.2%). Almost two-thirds of the West Hillhurst population are adults (64%) followed by youth (12.5%) and seniors (8.3%). In West Hillhurst, 13% of people are first-generation immigrants, and of those recently immigrating, 26.3% were born in the United States.

West Hillhurst park is an open green space (1.11 ha) that includes tall, well-established trees scattered throughout the park. Although not visible from within the park due to the presence of a utility building, a bridge at one end of the park provided a route for pedestrians and cyclists to cross over major arterial road (Memorial Drive) and joined with a pathway that runs along the Bow River. Within the park, an informal trail ran parallel to the chain-link fence separating the park from Memorial Drive (Additional file 6). This informal trail was frequently used by joggers headed toward the bridge intended for pedestrian and cyclist use, as well as by dog-walkers. The large, open grassy area that was well-maintained (e.g., regularly mowed) provided a suitable place for a range of recreational activities (Additional file 7). Dogs were frequently unleashed to roam and play fetch. This park had a playground in the northeast corner with benches and a garbage receptacle near the perimeter. The playground included three benches, a picnic table, two swing sets (one intended for preschoolers and one intended for older children), two animal figurines mounted on springs, a see-saw, a climbing apparatus, and a slide. At the top of one swing set was a sign that stated no dogs allowed within 20-meters of the playground. Graffiti was evident on the garbage can by the playground and on a box where the chain-link fence met a solid fence. In public consultations regarding the potential of formally designating an off-leash area within this park, local residents’ views were divided. Many participants appreciated bringing their dogs to the park, while other participants appreciated bringing their children to the playground and the park itself. Some but by no means all participants felt that dog-walking and childcare were compatible uses for this park [31].

#### Meadowlark park

Meadowlark was established in 1955, has a warped-grid street pattern, and is located in the southwest quadrant of the city. Meadowlark is a small community, with a population of 605 in 2011 (Table 2). In Meadowlark, 17.3% of people reside in rented dwellings. The neighbourhood has a median annual household income of $74,380 with 6.6% of persons living in low-income households – half of the percentage found for Calgary overall (14.2%). Among resident’s ≥25 years of age, 2.9% are unemployed and 36.9% have a high school diploma or less. Less than on quarter of residents are first-generation immigrants (24.8%), and among recent immigrants, two-thirds (66.7%) were born in the Philippines (Table 2).

Meadowlark park consists of multiple open green spaces (1.39 ha in total) separated by trees, shrubbery, and fences. Prior to the construction of this park in 2004, a school stood at this location. The school was removed and the park was created to allow for expansion and redevelopment of a major roadway immediately adjacent to the park. In the park, multiple connecting paved pathways designated for use by pedestrians and cyclists ran from the surrounding neighbourhood through the centre of the park and alongside the stone wall separating the park and neighbourhood from a major arterial road (Glenmore Trail). A dual-use path cut through the middle of the park, connecting the neighbourhood with an adjacent community and a major shopping mall. The adjacent community and major arterial road were separated from the park by a 10-foot high concrete fence which ran along the entire south side border of the park. Sidewalks were located on the northern perimeter of the park running adjacent to a local road. A chain-link fence enclosed an area up to the sidewalk on the northwest end. The park had landscaped features and a monument in the center of the park with surrounding benches (Additional file 8). Most grassed areas of the park were maintained, but were overgrown near a fenced area at the northwest corner of the park (Additional file 9). A public art installation had been constructed near the entrance from the surrounding neighbourhood to the park. It consisted of a brick and stone ‘tower’ topped by painted larks with plaques (painted to match the larks) that told the history of the park and surrounding neighbourhood. This public art installation was framed by attractive landscaping (e.g., pruned bushes, shrubs and small trees). Surrounding the public art installation were two benches, wrought-iron receptacles for garbage and litter, and street lamps. Graffiti was evident on multiple utility distribution poles along the park perimeter. Near the public art installation, there was a partially-fenced un-landscaped section (e.g., unmoved grass and overgrown with weeds), littered with construction debris. Chinook Shopping Centre could be seen from within the park, and this destination is readily accessible via designated pathways and the green space itself. The majority of residents in the neighbourhood of Meadowlark are Canadian-born and older in age than the City of Calgary average, yet older adults were rarely observed in the park. In public consultations regarding the potential designation of an off-leash area within this park, local residents indicated that they appreciated the park, and they complained about traffic and parking as issues that already compromised their use of this park and that off-leash designation could exacerbate [32].

### Descriptions of park use and physical activity between parks (quantitative observations)

#### Park use

Differences in time use patterns between the four parks were found (Table 3). For Taradale (72.5%), Martindale (82.1%), and Meadowlark (70.2%) parks, the majority of visitors were observed in the afternoon from 14:30–18:30 hrs, whereas for West Hillhurst, similar proportions of visitors were observed in the morning (49.1%) and afternoon (50.9%). The proportion of visitors to Taradale park was higher on Sundays (44.9%) compared with other days of the week and was significantly higher on Sundays than the other park sites (p < .05). The highest proportion of Meadowlark visitors was observed on Saturdays (48.3%), which was also significantly higher on Saturdays compared with the other parks sites (p < .05).

**Table 3:** **Proportion (%) of park users by different socio-demographic characteristics and usage patterns in the four study locations**

Characteristics	Taradale (a) (n = 167 observed)%	Martindale (b) (n = 184 observed)%	West Hillhurst (c) (n = 227 observed)%	Meadowlark (d) (n = 205 observed)%
**Gender**				
Female	35.3	38.6	44.8	42.4
Male	64.7	61.4	53.7	57.6
Unknown**	0.0	0.0	1.3	0.0
Age group*				
Child/teenager	23.4^b, c, d^	58.7^a, c, d^	35.7^a, b^	33.2^a, b^
Adult	76.6	41.3	64.3	66.8
Group (other people)*				
Alone	46.7^c^	42.9^c^	36.1^a, b, d^	50.2^c^
With another person	53.3	57.1	63.9	49.8
Time of day*				
Morning (8:30-12:30)	27.5^b, c^	17.9^a, c, d^	49.1^a, b, d^	29.8^b, c^
Afternoon (14:30-18:30)	72.5	82.1	50.9	70.2
Day of week*				
Weekday	31.1^b^	44.0^a, d^	35.2^d^	24.4^b, c^
Saturday	24.0^c, d^	25.5^c, d^	37.8^a, b, d^	48.3^a, b, c^
Sunday	44.9^b, c, d^	30.4^a^	27.0^a^	27.3^a^
Activity type inside park*				
Sedentary	1.8^c, d^	1.1^c, d^	15.9^a, b, d^	11.2^a, b, c^
Walking	28.1^b, c, d^	42.4^a, c, d^	7.0^a, b, d^	58.5^a, b, c^
Dog-related activity	36.5^b, d^	24.4^a, c, d^	41.9^b, d^	4.9^a, b, c^
Jogging/running	2.4	4.3	4.0	2.9
Cycling	28.1^c^	25.0^c^	3.1^a, b, d^	25.9^c^
Playing	4.2^c^	6.0^c, d^	33.5^a, b, c^	2.4^b, c^
Observed for 2-minutes*	94.6^b, d^	88.6^a, c, d^	95.7^b, d^	43.4^a, b, c^

*Statistically significant differences for category among parks based on Pearson’s Chi-Square; ^a^Significantly different from Taradale (p < .05); ^b^Significantly different from Martindale (p < .05); ^c^Significantly different from West Hillhurst (p < .05); ^d^Significantly different from Meadowlark (p < .05)**Unknown due to unidentifiable sex of infant in stroller. Note for activity type, multiple activities could be reported for a single case.

#### Park visitor characteristics

In the four parks, a combined total of 783 visits were observed. Overall, West Hillhurst (n = 227), followed by Meadowlark (n = 205), Martindale (n = 184), and Taradale (n = 167) had the most observations of park use (Table 3). The majority of those observed in Taradale (94.6%), Martindale (88.6%), and West Hillhurst (95.7%) parks were observed for a full two minutes. By contrast, less than half of the observations in Meadowlark took two minutes (43.4%). Observations lasting fewer than two minutes included visitors who walked or cycled through the parks en route elsewhere. The majority of individuals observed in Meadowlark park were either walking or cycling along pathways crossing the park. Males were more likely to be observed in the parks than females, with no statistically significant differences between the four parks. Compared with the other sites, those observed in Taradale park were more likely to be adults (76.6%, p < .05) reflecting the lack of facilities for children’s activities. For Martindale park, those observed tended to be youth as opposed to adults or children, again reflecting a lack of facilities to support play amongst youngsters (58.7%, p < .05). For West Hillhurst and Meadowlark, similar proportions of adults and youth were observed. Compared with the Taradale, Martindale, and Meadowlark parks, the proportion of those visiting alone was significantly lower in West Hillhurst (36.1%, p < .05). Children and adult caregivers were frequently observed using the West Hillhurst park facilities (i.e., the playground).

#### Park activity types

The majority of park visitors participated in only one activity whilst being observed, with fewer undertaking multiple activities (10.5%). The two most popular activities across all parks included walking (33.3%) and dog-related physical activity (26.7%) (e.g., walking or running with a dog, throwing the ball) (Table 3). Walking (without a dog) was the most popular activity in Meadowlark (58.5%, p < .05) compared with the other three sites, while walking was less common in West Hillhurst (7%, p < .05) compared with the other sites. Compared with Martindale and Meadowlark, a higher proportion of dog-related activity – the majority of which included dog-walking – was observed in West Hillhurst (41.9% p < .05) and Taradale (36.5%, p < .05), respectively. The proportion of joggers/runners was not significantly different between the parks (2.4 to 4.3%). At least one-quarter of visitors in the Taradale (28.1%), Martindale (25%), and Meadowlark (25.9%) parks were observed cycling compared with significantly fewer cyclists observed in West Hillhurst park (3.1%, p < .05). The lower levels of walking and cycling observed in West Hillhurst park relative to the other parks likely reflect the location of the pathway in relation to the open space. The pathway which was adjacent to one end of the park space and the park space itself was not generally used as a corridor linking between destinations. Play activity (not dog related) was significantly higher in West Hillhurst (33.5%, p < .05) compared with the other parks (range 2.4 to 6.0%), which could reflect the low levels of pedestrian and cyclist traffic through the park. In addition, West Hillhurst park included the highest proportion of sedentary activity (15.9%) followed by Meadowlark (11.2%), Taradale (1.8%), and Martindale (1.1%) parks.

## Discussion

Among the four park study sites, we found variations in the patterns of use, characteristics of users, and the types of activities undertaken. In general, the patterns of use in all four parks combined reflected findings regarding the gender and age characteristics of urban park visitors elsewhere [33–36]. However, contrary to previous evidence [18, 19, 34, 37], we found that sedentary activities were not the most common park activity. Rather, walking and dog-walking were the most common activities for our sample of parks. Our findings also suggest that patterns of park activities are associated with the physical attributes within each of the parks and the surrounding neighbourhoods. Few other studies have integrated multiple quantitative and qualitative approaches (i.e., structured observation, fieldnotes, and photography) to interpret the interrelationships between the characteristic of parks and surrounding neighbourhoods and park use and activity [38].

Despite being unable to collect data for the entire day, we found that patterns of park use were consistent with patterns of use found elsewhere [33, 34, 39], whereby that the majority of visits take place in the afternoons and evenings. Patterns of weekday and weekend (Saturday and Sunday) use differed between parks but not in a consistent way. Across the four parks, male visitors were more common than female visitors, while for three of the four parks, users were more likely to be adults than children or youth – a pattern that is consistent with evidence collected using systematic observation [33–36]. While we were unable to quantify the interaction between gender and activity types within the parks due to sample size, elsewhere men have been found to be more physically active within park settings than have women [19, 35, 36]. This pattern is similar to differences in physical activity found between men and women in population-based studies [40, 41]. Creating more opportunities for physical activity within parks could contribute to more park-based physical activity among women, thereby reducing gender disparities in physical activity. To encourage more park visits and more park-based physical activity among women, design and management strategies could include improvements associated with safety (e.g., lighting and surveillance) as well as greater opportunities for social interaction [9].

Park visitors are often more likely to be adults than children or youth [18, 19, 34, 42]. In one of our parks (i.e., Martindale) however, over one-half of all users observed were children or youth. This park did not include physical attributes, such as playgrounds, typically associated with children’s use and physical activity within parks [21]. Moreover, within this same park, participation in “play” activity was low compared with other activities which is noteworthy as we expected to see more “play” in settings with high numbers of children. Our qualitative data suggest that the youth who visited this park were unaccompanied by adults and that they visited a central secluded wooded area to ‘hang-out’ and socialize. Furthermore, monuments within the Martindale park were used for play activity, such as climbing. This finding stands in contrast with the West Hillhurst park, which included a playground, where one-third of visitors were children or teenagers, and where one-third of activity observed was “play”. Our findings suggest that playgrounds, while encouraging children to be physically active, are not the only reason why children visit parks. For older children and teenagers, park attributes that provide places for friends to meet and socialize are likely just as important [9]. Introducing age-appropriate playground equipment, creating and maintaining sports fields and courts and open spaces, as well as the provision of areas for socializing that balance privacy with safety, might encourage more children and teenagers to be physically active by visiting parks [9, 33].

Sedentary behaviour is commonly observed in parks [18, 19, 34, 37]. Kaczynski et al. [19] found that just over half of observed park users participated in sedentary activity while Floyd et al. [18] and Cohen et al. [37] found that almost two-thirds of park users were sedentary. Contrary to this evidence, our findings suggest that for the four parks included in our study, sedentary activity was less common than other activities. Nevertheless, observed participation in sedentary activity differed between the parks and the reasons for these differences are in part explained by the qualitative data. Located within Taradale and Martindale parks are storm water drains and pools of stagnate water that facilitate mosquito breeding, which is particularly severe during the early months of the Calgary summer. The mosquito populations within these two parks were particularly numerous during the data collection (“unbearable” as pointed out by LB and KS while conducting the observations in these locations), likely deterring people from spending extended periods of sedentary time (i.e., sitting, standing, remaining stationary) within these locations. Furthermore, the parks were relatively small in area and the layout of the open space was not particularly suitable for organized or unorganized sports activities, which often attract (sedentary) spectators [37]. Seating was also entirely absent (in Martindale) or in short supply (in Taradale). Nevertheless, even those observed to be sedentary in parks likely derived some health benefit from the physical activity accumulated whilst traveling to and from the park. Active transportation is a common mode of transportation for reaching public recreational opportunities [43]. Creating and redesigning parks to include attributes and amenities that support both passive and active pursuits, therefore, could increase levels of physical activity in urban populations [44, 45].

People may visit parks more frequently when accompanied [46, 47]. We found that almost two-thirds of park visitors were with another person and that about one-half of children visiting parks were accompanied by parents or guardians. Compared with other parks, West Hillhurst included the highest proportion of group visits. Because of its playground, “playing” was also the most common activity in West Hillhurst park relative to the other parks. Together, these findings might suggest that some parents and caregivers are physically active while accompanying their children to the playground, but are themselves inactive in parks while their children play. The physical attributes within this park including benches and shade close to the playground and the limited amount of pathway might facilitate sedentary activity among these caregivers. Besenyi et al. [21] found that for adults and seniors higher levels of park-based physical activity energy expenditure occurred on paths and courts compared with other areas (open spaces, picnic areas), while for children, higher levels of park-based physical activity energy expenditure occurred in playgrounds. Floyd et al. [33] found that less shade was associated with a greater likelihood of park users walking and participating in vigorous-intensity activity versus sedentary behaviour. Shores and West [22] also found sedentary activities to be more common in parks with shelter and picnic areas. One suggestion for park planners might be to include attributes that will support physical activity for adults (e.g., fitness zones, walking paths) near playgrounds so parents or caregivers can still supervise their children while they are physically active themselves.

Dogs, in our study, contributed positively to patterns of physical activity. After walking without a dog, dog-walking and other dog-related pursuits, such as playing fetch, were the most commonly observed activities. These findings extend a growing body of research in health promotion linking dog-walking with physical activity [48]. This line of inquiry is highly relevant to population-level patterns of physical activity because in Canada, dogs reside in one out of every three urban households [49], and similar statistics have been reported in other Western countries [50, 51]. Dog-walking is also becoming a common form of physical activity in some non-Western countries, such as China and Japan [52, 53]. Nevertheless, high levels of dog-ownership can also impact negatively on physical activity for both dog-owners and non-dog-owners, given that ill-controlled dogs and litter from dog-waste can deter people from visiting parks [51], so policies and management strategies need to take dog and owner behaviour into account.

Our method of systematic observation differed from methods used elsewhere (i.e., SOPARC, SOPLAY). Unlike other approaches, we attempted to record park user information and activities for the entire park and not for separate activity zones within the park. This approach was facilitated because of our focus on neighbourhood parks that were typically smaller than other open spaces (e.g., community parks, regional/national parks, sports fields). Because we centered our observations on persons, not on multiple activity areas in the same park, double counting of visitors was minimized. When multiple activity areas serve as the basis for observations, any visitors who moved between activity areas during observation periods could be double-counted [39]. Whenever possible, we observed each park user for a full two minutes so as to document multiple activities (if undertaken within such a short time-frame) during the same park visit. The time frame of two minutes was selected to balance the need to capture the range of activities undertaken by a single individual and the need to include as many cases as possible. In the Taradale, Martindale, and West Hillurst parks, most visitor observations lasted for two minutes. In Meadowlark however, less than one-half of all visitors were observed for two minutes. Meadowlark included a short pathway that connected a residential area and large mall both of which were surrounded by major roads with high volume traffic. Many walkers and cyclists observed in the Meadowlark park were passing through en route to other destinations, thus the fact that fewer visitors were observed for the entire two minutes reflects the activity patterns within, and the design of, the park. During times when the volume of visitors was low, observation of visitors may have begun immediately as they entered the park – thus their activity would likely have been recorded as walking or cycling but underestimating other types of activity (e.g., sedentary activity or play). Similarly, visitors who were already in the park and were being sedentary, playing, or undertaking dog-related activity likely entered the park via walking or cycling, so our results may underestimate walking and cycling. Whereas our findings are a cross-sectional representation of summer park activities in four parks only, they suggest that the relationships between park policies and physical activity are complex.

Despite limitations, our case study approach provided a rich description of the activities and settings and their interaction within the four parks. Indeed, such a detailed description would not have been possible using a single methodological approach or data source. The unique findings from our case study highlight that while policy-related physical attributes in some parks appear to encourage certain types of behaviour (e.g., the presence of playgrounds and frequency of play amongst children), in other parks, the relationship between context and behaviour was less than straight-forward. Thus, the physical and social characteristics as well as the history of the community in which a park is situated are potentially important influences on park use. The case study approach might provide useful information for park developers and planners in creating or modifying parks, for example, to encourage specific types of activities or to encourage more use among particular types of visitors [6]. Furthermore, this approach is highly relevant to innovations in park planning and management, which often begin with pilot or demonstration projects that require consideration of ambient policy, sociocultural and geographical contexts [6].

## Conclusions

Park attributes and the surrounding neighbourhood social characteristics are important for determining the types of park-based activities visitors undertake and the socio-demographic profile of visitors. The desire to sit outside, surrounded by nature, might be the very reason people visit parks, yet this desire could still contribute to physical activity levels by prompting people to walk or cycle, to and from parks. Planners and mangers of parks should consider the potential impacts that physical and symbolic attributes may have on visits and they should look to maximize attributes that encourage park visitors to be physically active.

## Author information

Gavin McCormack, PhD, is an Assistant Professor in the Department of Community Health Sciences in the Cumming School of Medicine, Adjunct Associate Professor in the Faculty of Environmental Design, and a member of the Institute of Public Health at the University of Calgary. Melanie Rock, PhD, is an Associate Professor in the Department of Community Health Sciences (primary appointment) in the Cumming School of Medicine and is Scientific Co-Director of the Population Health and Inequities Research Centre in the Institute for Public Health at the University of Calgary. Kenda Swanson is a Master of Science candidate in the Department of Community Health Sciences, Cumming School of Medicine, at the University of Calgary and graduate of the O’Brien Centre Bachelor of Health Sciences program (University of Calgary). Lindsay Burton is a Master of Science candidate in Interdisciplinary Studies at the University of British Columbia and a graduate of the O’Brien Centre Bachelor of Health Sciences program (University of Calgary). Alessandro Massolo, PhD, is an Assistant Professor of Wildlife Health Ecology in the Department of Ecosystem and Public Health in the Faculty of Veterinary Medicine at the University of Calgary.

## Electronic supplementary material

Additional file 1: **Photograph 1.** (JPG 4 MB)

Additional file 2: **Photograph 2.** (JPG 3 MB)

Additional file 3: **Photograph 3.** (JPG 4 MB)

Additional file 4: **Photograph 4.** (JPG 4 MB)

Additional file 5: **Photograph 5.** (JPG 4 MB)

Additional file 6: **Photograph 6.** (JPG 4 MB)

Additional file 7: **Photograph 7.** (JPG 4 MB)

Additional file 8: **Photograph 8.** (JPG 3 MB)

Additional file 9: **Photograph 9.** (JPG 4 MB)
