# Supplementary figures and images for: Physical activity patterns in urban neighbourhood parks: insights from a multiple case study
Source: BMC Public Health. 2014 Sep 17;14:962. doi: 10.1186/1471-2458-14-962 (PMC4247115; doi:10.1186/1471-2458-14-962)

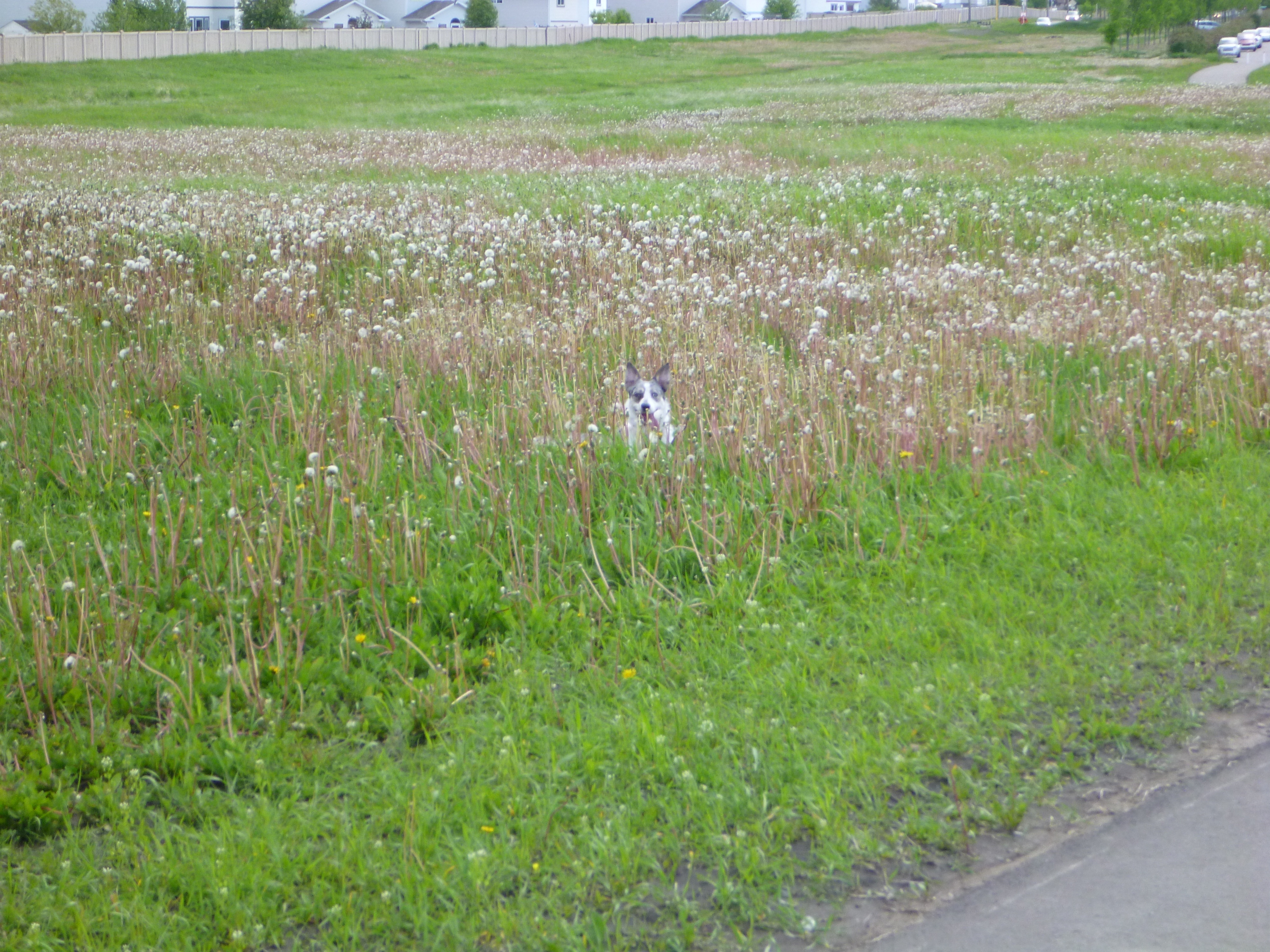

Supplement: Supplementary file 1 — Additional file 1: Photograph 1. (JPG 4 MB) [file 12889_2014_7300_MOESM1_ESM.jpg]

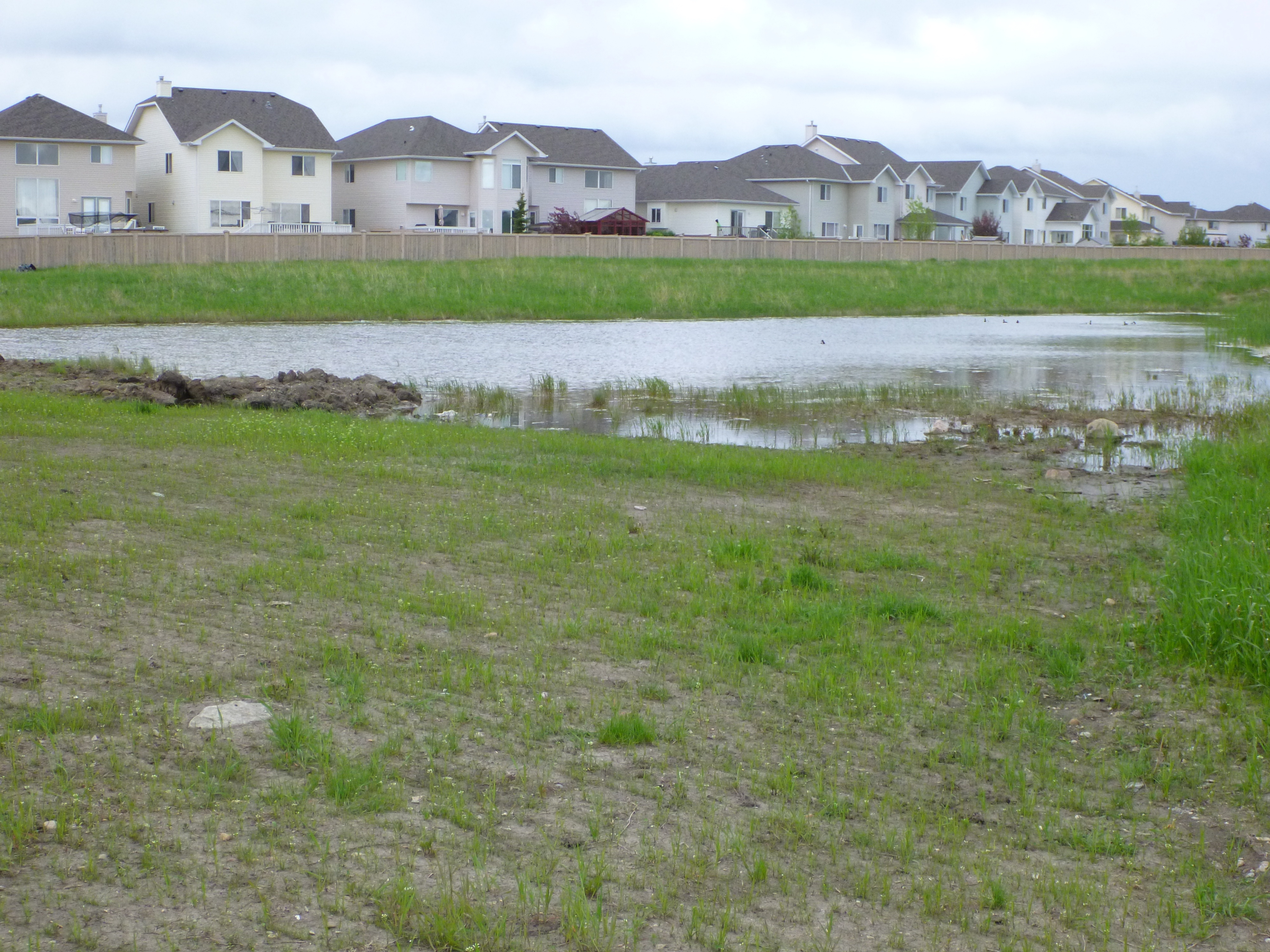

Supplement: Supplementary file 2 — Additional file 2: Photograph 2. (JPG 3 MB) [file 12889_2014_7300_MOESM2_ESM.jpg]

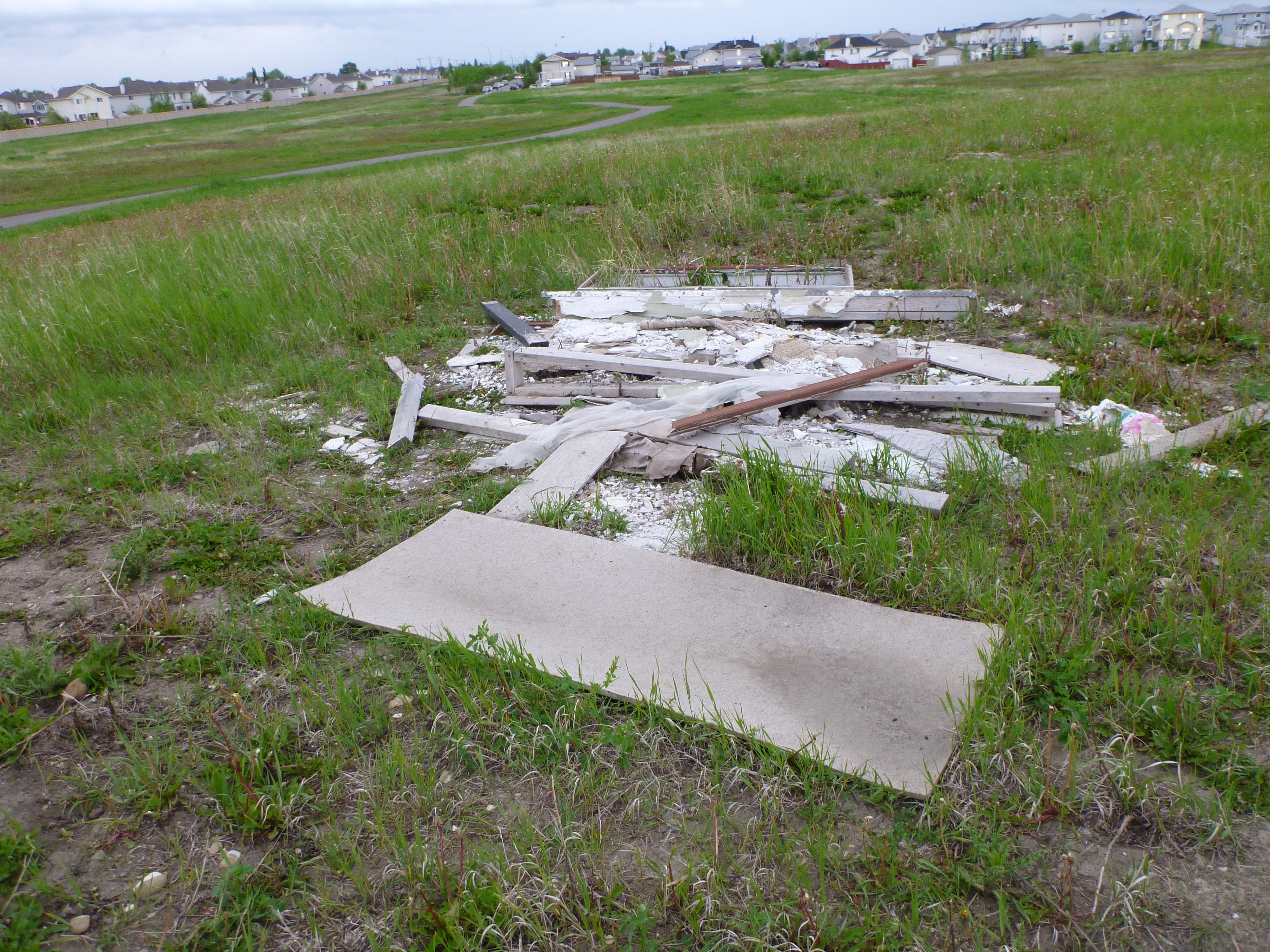

Supplement: Supplementary file 3 — Additional file 3: Photograph 3. (JPG 4 MB) [file 12889_2014_7300_MOESM3_ESM.jpg]

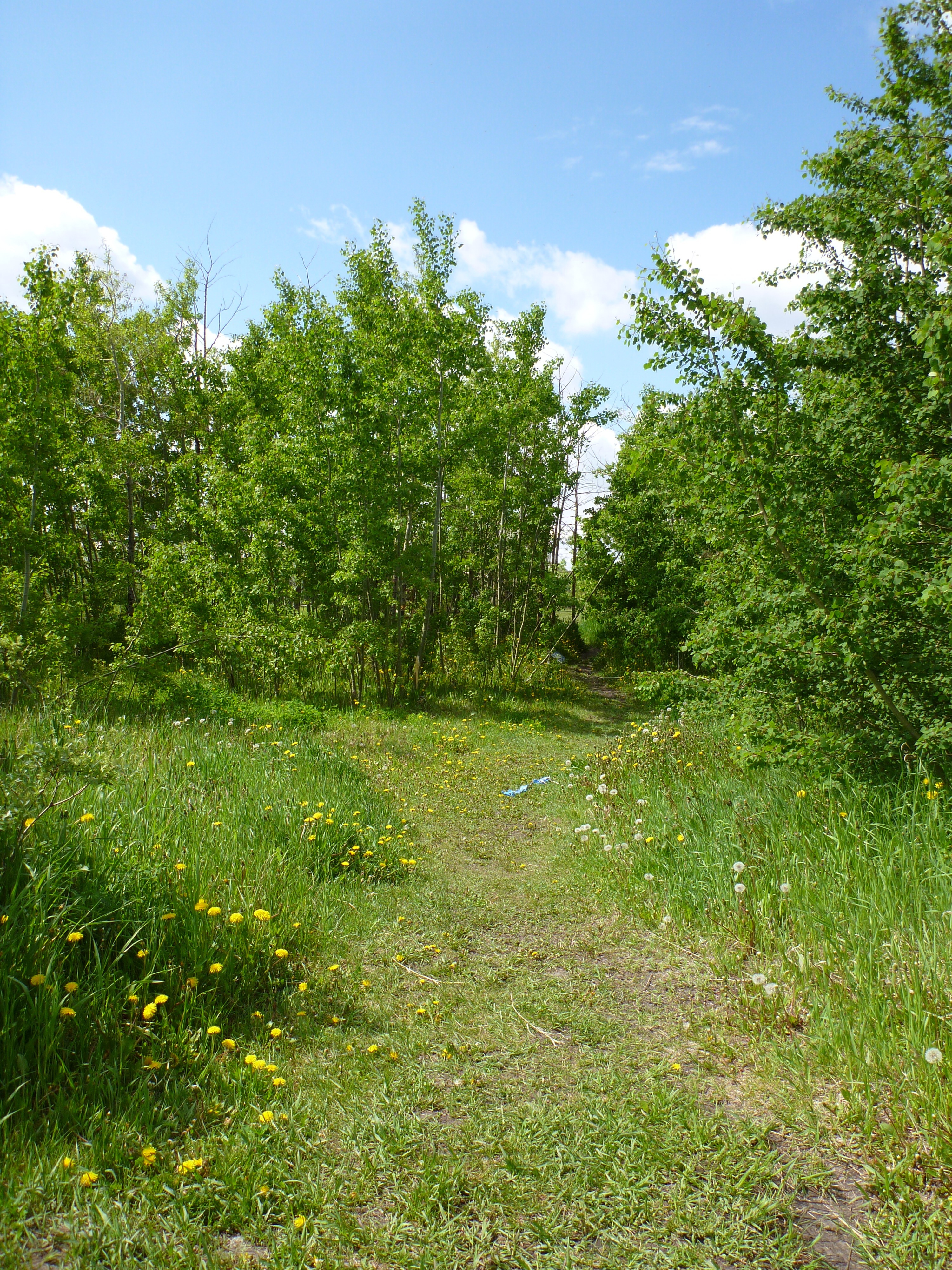

Supplement: Supplementary file 4 — Additional file 4: Photograph 4. (JPG 4 MB) [file 12889_2014_7300_MOESM4_ESM.jpg]

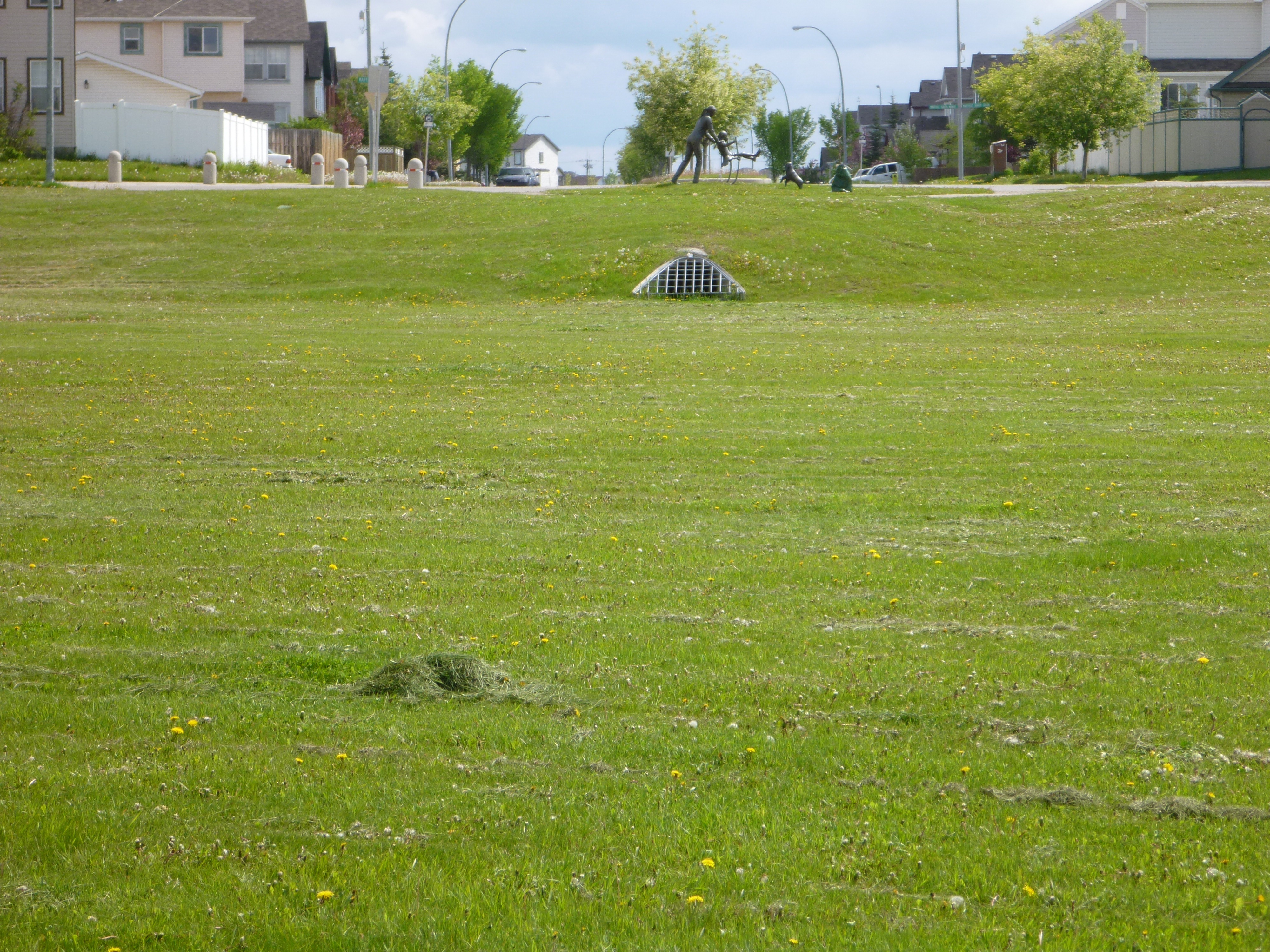

Supplement: Supplementary file 5 — Additional file 5: Photograph 5. (JPG 4 MB) [file 12889_2014_7300_MOESM5_ESM.jpg]

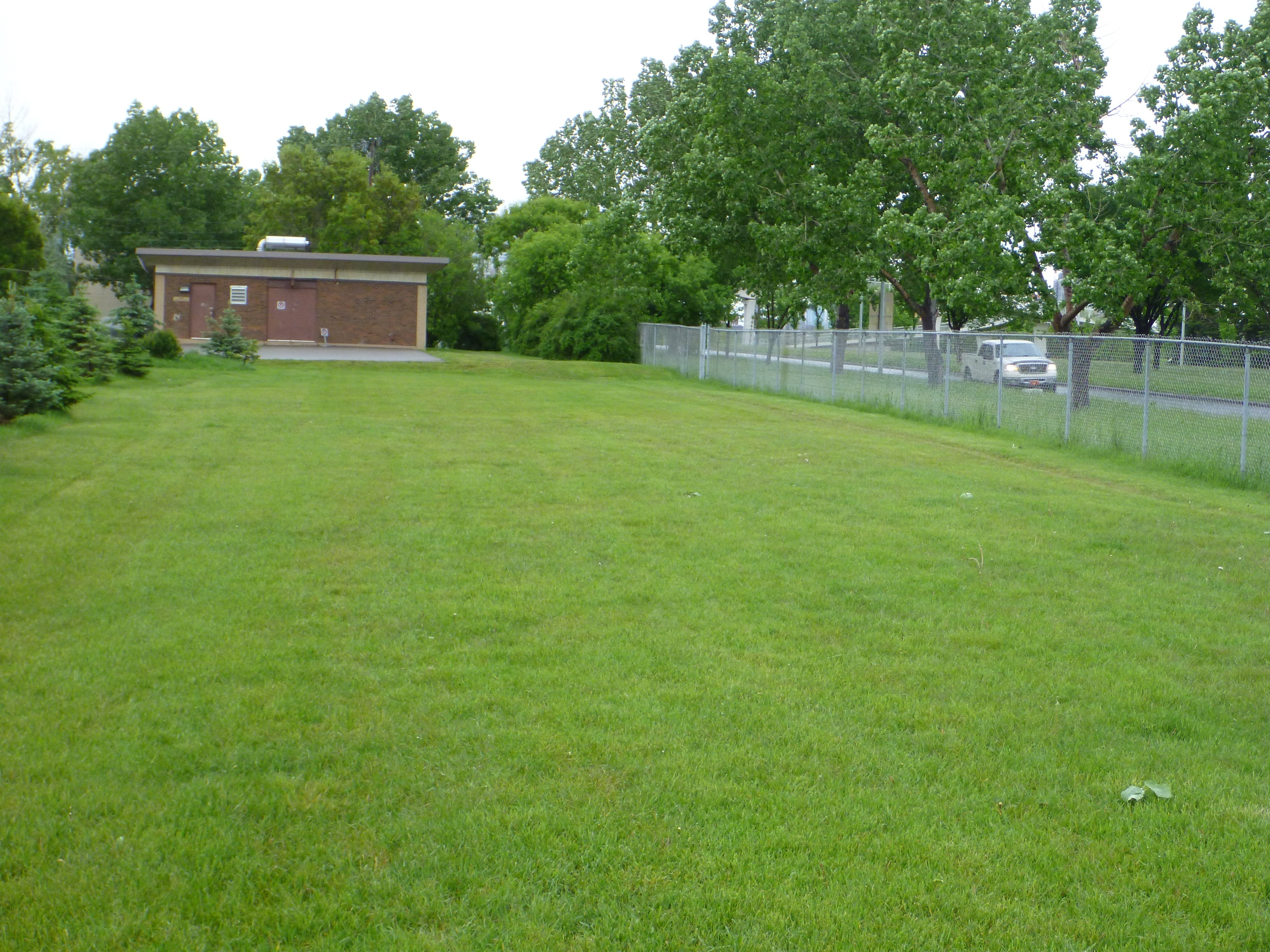

Supplement: Supplementary file 6 — Additional file 6: Photograph 6. (JPG 4 MB) [file 12889_2014_7300_MOESM6_ESM.jpg]

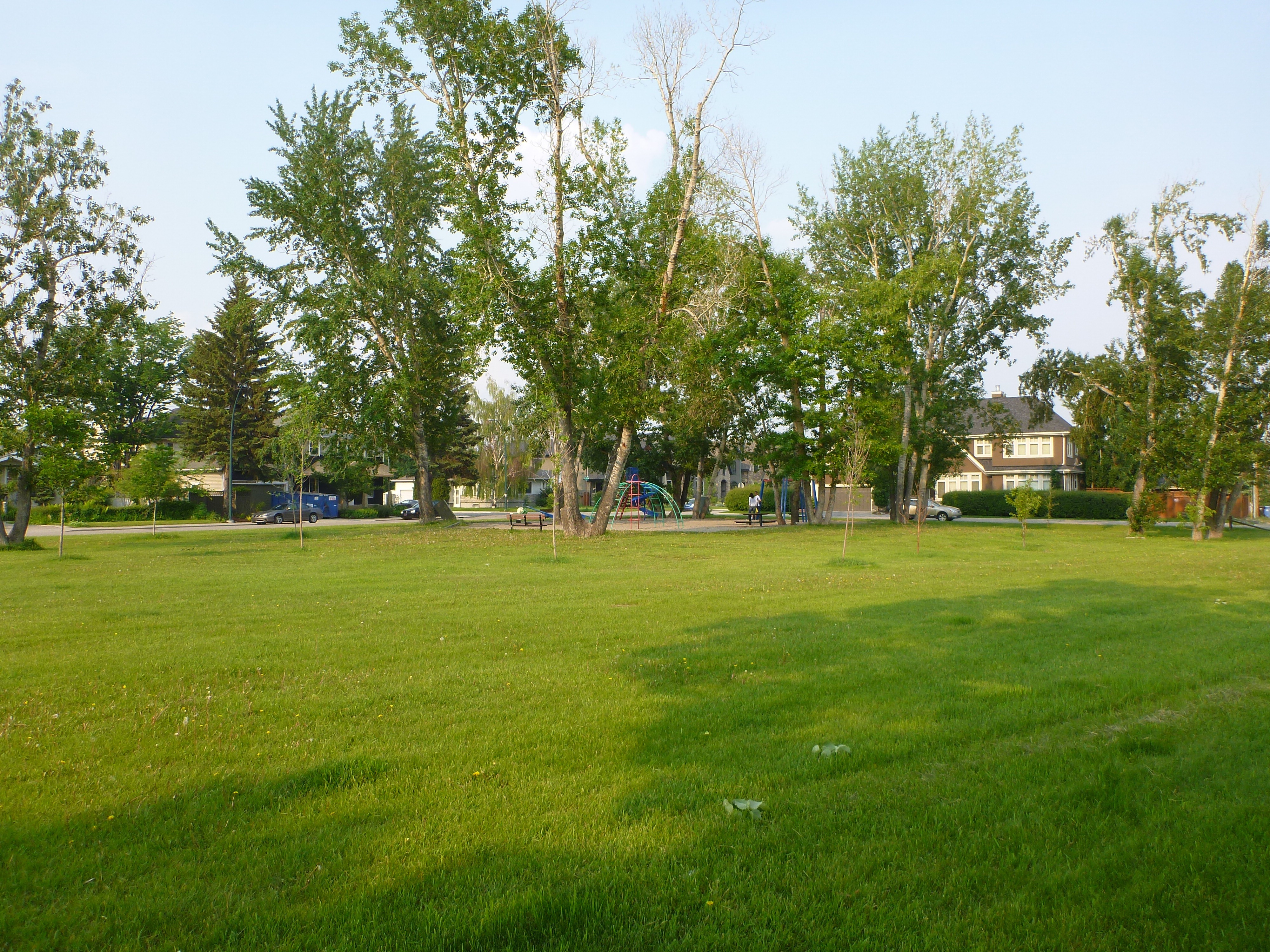

Supplement: Supplementary file 7 — Additional file 7: Photograph 7. (JPG 4 MB) [file 12889_2014_7300_MOESM7_ESM.jpg]

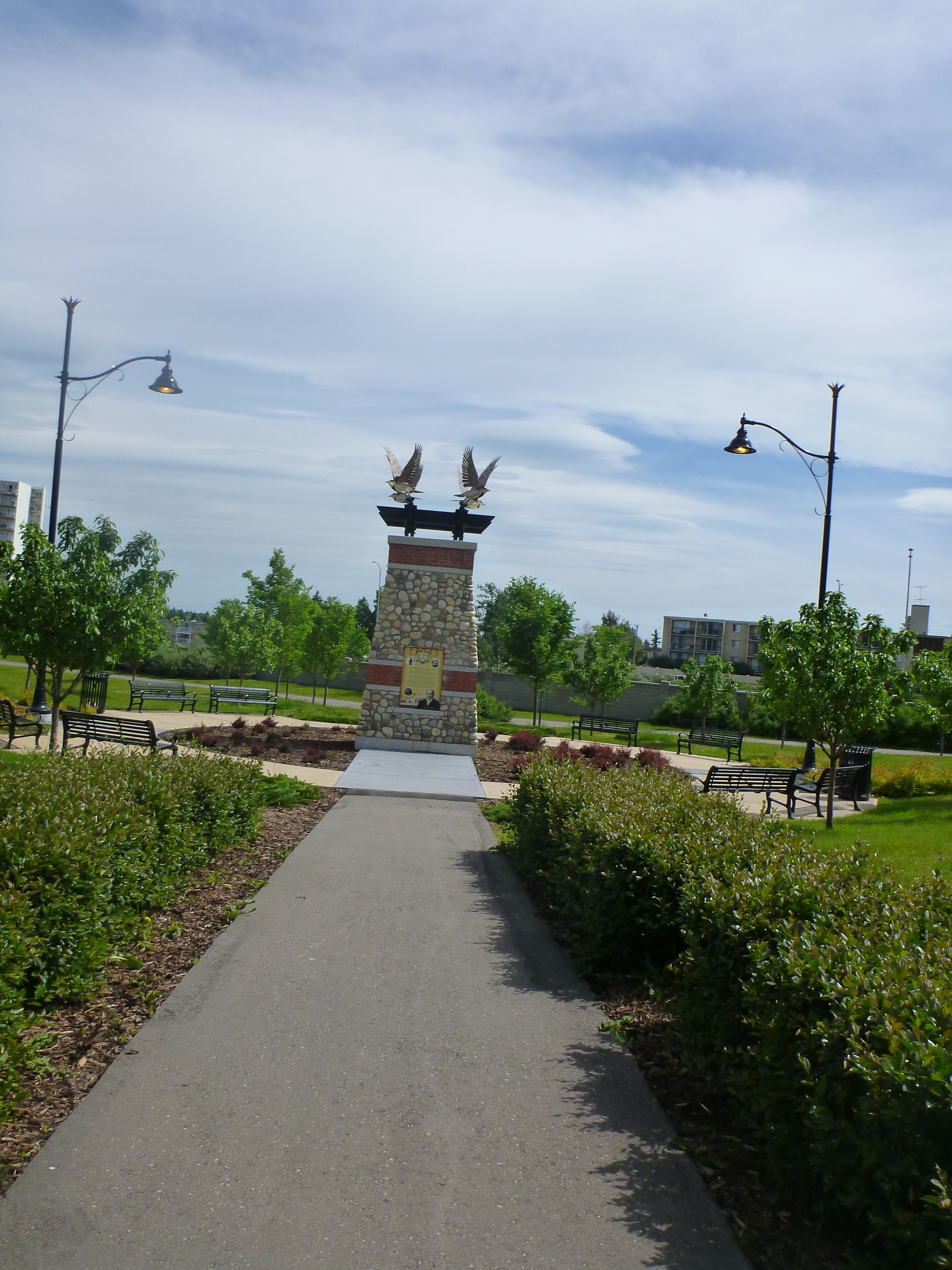

Supplement: Supplementary file 8 — Additional file 8: Photograph 8. (JPG 3 MB) [file 12889_2014_7300_MOESM8_ESM.jpg]

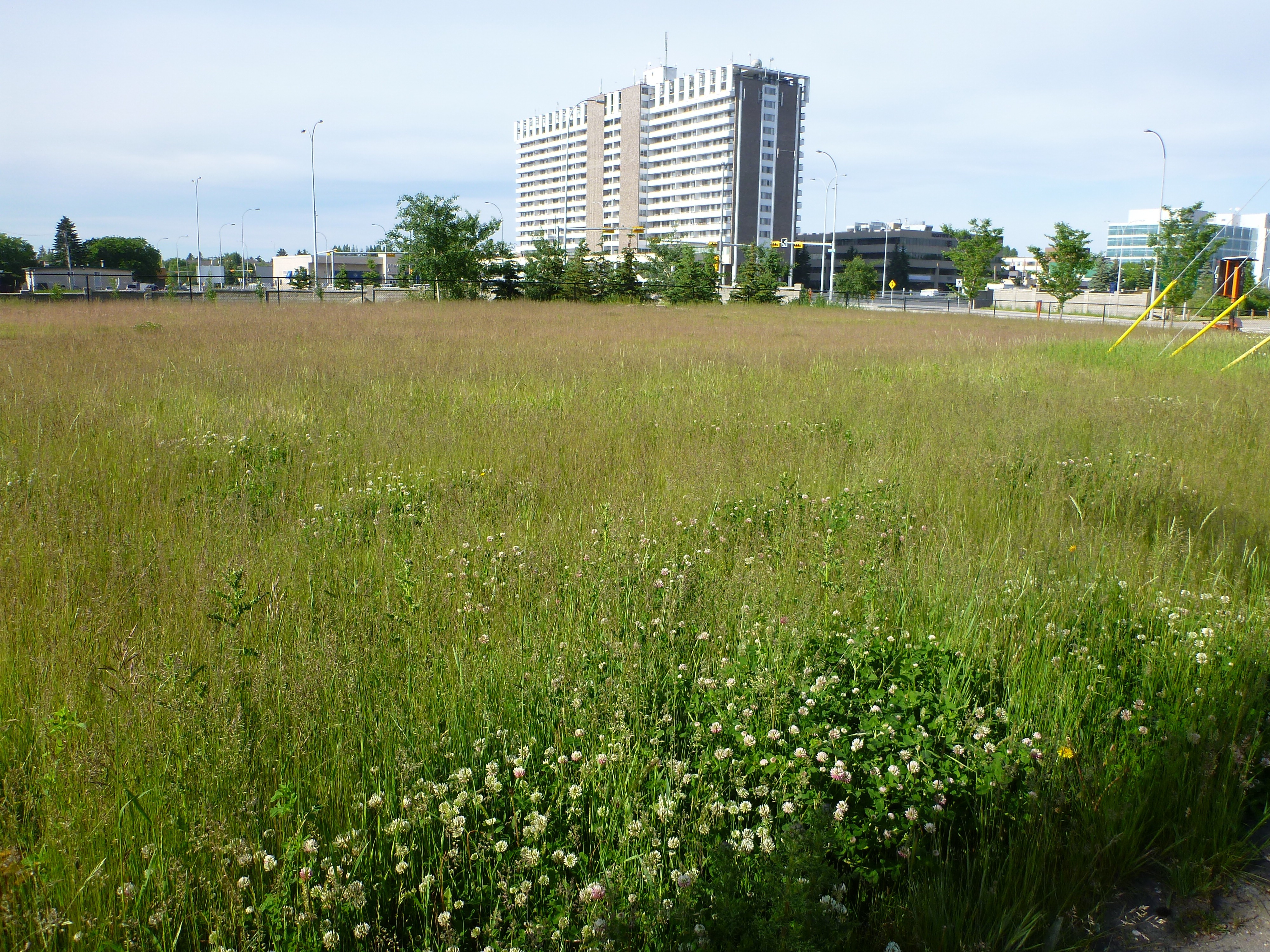

Supplement: Supplementary file 9 — Additional file 9: Photograph 9. (JPG 4 MB) [file 12889_2014_7300_MOESM9_ESM.jpg]
